# TP53 and RET may serve as biomarkers of prognostic evaluation and targeted therapy in hepatocellular carcinoma

**DOI:** 10.3892/or.2022.8411

**Published:** 2022-09-19

**Authors:** Song Ye, Xin-Yi Zhao, Xiao-Ge Hu, Tang Li, Qiu-Ran Xu, Huan-Ming Yang, Dong-Sheng Huang, Liu Yang

Oncol Rep 37: 2215–2226, 2017; DOI: 10.3892/or.2017.5494

Subsequently to the publication of the above article, the authors have realized that a couple of clerical errors were made when writing the article, and wish to correct these errors in a corrigendum statement. First, in the Materials and methods section on p. 2216, the final sentence of the ‘*Immunohistochemistry and tissue microarray*’ subsection, the authors wish to add a further definition, so that the text reads as follows (changes highlighted in bold): ‘**The positive expression of RET was defined as ≥5% staining of a tumor section;** high expression was defined as ≥40% staining of a tumor section, and low expression as <40%’. Secondly, in the Results section, ‘*Mutation frequency of each gene distributed in 4 biological categories*’ subsection, p. 2220, right-hand column, second paragraph, line 17, the sentence written here should have read as follows: ‘The group with the **positive** expression of RET included 28.9% (26/90) of the patients, **and 4 of these patients were defined as high expression**’.

The authors are grateful to the Editor of *Oncology Reports* for allowing them this opportunity to publish a corrigendum, and apologize to the readership of the journal for any inconvenience caused.

